# Risk factors associated with gonorrhea and chlamydia transmission in selected health facilities in Ghana

**DOI:** 10.1186/s12879-019-4035-y

**Published:** 2019-05-16

**Authors:** Helena Dela, Naiki Attram, Eric Behene, Selassie Kumordjie, Kwasi Kennedy Addo, Edward Owusu Nyarko, Nicholas N. A. Kyei, John Nii Ayite Carroll, Cynthia Kwakye, Christopher Anthony Duplessis, Nehkonti Adams, Eric Garges, Andrew Gordon Letizia

**Affiliations:** 1 Noguchi Memorial Institute for Medical Research (NMIMR), Legon, Ghana; 2 US Naval Medical Research Unit 3 Ghana Detachment, Accra, Ghana; 3 37 Military Hospital, Accra, Ghana; 4 Airforce Medical Center, Takoradi, Ghana; 5 Ghana Health Service, Accra, Ghana; 6 Naval Medical Center, San Diego, USA; 7 Silver Spring, USA

**Keywords:** Gonorrhea, Chlamydia, Ghana, Risk factors, STIs

## Abstract

**Background:**

Understanding the underlying epidemiology that shapes *Neisseria gonorrhoeae* (GC), and *Chlamydia trachomatis* (CT) infections can contribute to data driven policies directed towards curbing the proliferation of these pathogens in Ghana. Information on the symptoms and risk factors for STIs will help to identify high-risk individuals which will in turn inform STI syndromic management and tailor the use of public health resources.

**Methods:**

Participants were from 4 military clinics and 1 civilian STI clinic in Ghana and eligible if they had symptoms suggestive of STI. First void urine samples were collected and tested with Nucleic Acid Amplification Test (NAAT). A structured questionnaire was administered to all participants. Multivariate logistic regression identified factors associated with infection, separately for NG and for CT and for men and women.

**Results:**

A total of 950 patients, 58% of whom were females were enrolled, 28% had gonorrhea and 11% had chlamydia with more males testing positive than females. Reported symptoms that were more common among patients who tested positive for gonorrhea were painful urination and urethral discharge (all *P* values < 0.05). Additionally, multiple sexual partners and alcohol use were statistically associated with higher rates of gonorrhea in males while only the frequency of condom use was associated with gonorrhea for females. None of the symptoms or risk factors except marital status was associated with testing positive for chlamydia.

**Conclusion:**

Identifying these symptoms and risk factors help inform health care delivery systems for STIs in Ghana. Furthermore, men and women presenting with these symptoms and risk factors are a prime target for public health education campaigns, aimed at curbing the spread of gonorrhea and chlamydia infections.

## Background

More than one million sexually transmitted infections (STIs) are acquired globally everyday [1]. These include common treatable STI pathogens such as *Treponema pallidium*, *Neisseria gonorrhoeae*, *Chlamydia trachomatis* and *Trichomonas vaginalis.* The largest disease burden occurs in developing nations within South and South-East Asia, followed by Sub-Saharan Africa [2]. *N. gonorrhea* and *C. trachomatis* comprise the most common bacterial STIs globally and can present as co-infections. This is of particular concern in Sub-Saharan Africa since both of these infections can potentiate HIV infection exacerbating the epidemic on the continent [3, 4]. In females, symptoms can include abnormal vaginal discharge, dysuria, dyspareunia, hematuria, and lower abdominal pain. However, these symptoms are frequently absent, contributing to delayed treatment and further transmission. Untreated, these infections predispose females to ectopic pregnancy and can cause Pelvic Inflammatory Disease (PID), infertility, and chronic pelvic pain as well as blindness and pneumonia in neonates. Males typically present with penile discharge and/or dysuria, which may lead to complications including penile stricture, epididymoorchitis, and sterility. These chronic complications can have a profound negative effect on the individual and the public health of a nation. Gonorrhea and chlamydia significantly contribute to elevated disability adjusted life years (DALYs) [5–7].

The Ghanaian Ministry of Health guidelines for STI management utilizes the WHO recommended syndromic approach to patient evaluation and treatment due to the lack of cost-effective laboratory tests [8–10]. Syndromic management is an approach in which clinical algorithms for commonly presenting signs and symptoms (e.g., painful urination and urethral discharge) are used to define cases [11]. In low resource settings, the syndromic approach can be used as a tool to aid diagnosis, and treat STIs.

Data on the overall incidence of gonorrhea and chlamydia in individuals presenting with symptoms consistent with STIs in Ghana is limited. Most saliently *N. gonorrhea* and chlamydia risk factors may be vastly different in Ghana than in other geographical locales (including in Sub-Saharan Africa) as they are influenced by economic factors, indigenous norms, and sociocultural influences that are different than other areas, especially the developed-world. Sexual and reproductive healthcare in Ghana will improve with more efficient case detection and better prevention strategies [9, 12].

The aim of this study was to identify factors associated with gonorrhea and chlamydia infection and determine the prevalence of both diseases in symptomatic individuals. This information will provide insight on which populations are most at risk of acquiring gonorrhea or chlamydia allowing for data driven decisions concerning which population groups should be targeted for use of limited diagnostic capacity, screening programs, treatment strategies, and targeted public health educational campaigns within Ghana.

## Methods

Surveillance was performed in two military clinics in Takoradi, one in Sekondi, one at the 37 Military Hospital, as well as the civilian Adabraka STI clinic in Ghana. Patients seeking care at the selected clinics with symptoms consistent with urethritis or cervicitis between June 2012 and March 2016 were approached for consenting and enrollment. Inclusion criteria included urethral discharge or dysuria in men as well as vaginal discharge, intermenstrual bleeding, or abdominal pain in women. Exclusion criteria were volunteers unable to provide consent, patients less than 12 years of age, had already participated in the study, or those without an STI syndrome or suspicion of gonorrhea.

Eligible patients were made to provide a written informed consent before enrollment. During the consenting process, all eligible patients provided a urine specimen and completed a structured questionnaire [13–15] and then had the option of providing a urethral/endocervical swab for culture as well. Details of the laboratory methods used in this study have been described elsewhere [16].

Ethical approval was received from the Naval Medical Research Unit Number 3, Cairo, Egypt with an approval number of NAMRU3.2012.0007.

### Laboratory analysis

Nucleic Acid Amplification Testing (NAATs) was performed for both gonorrhea and chlamydia on DNA extracts from urine samples. The LightMix® kit was used to detect both *Neisseria gonorrhea* and *Chlamydia trachomatis* DNA (TIB MOLBIOL, Berlin, Germany) using the LightCycler 480II (Roche Diagnostics, Germany) according to the manufacturer’s instructions.

### Statistical analysis

Preliminary analysis was done using basic descriptive statistics in which frequency and percentage were applied to describe the laboratory outcome (gonorrhea and chlamydia infection) and independent variables. Chi-square and Fishers exact test were used where appropriate to determine the association between the outcome and independent variables stratified by gender. The significance level was set at a *p*-value < 0.05. Variables which had p-value < 0.2 were selected and fitted into a multivariable logistic regression. Final model was determined using a stepwise backward selection method, removing the least significant variable and re-computing until all remaining variables were significant (*p* < 0.05). Five variables were forced into the final model after they were dropped during the variable selection process because they were considered as a priority potential predictor for STI infection. These were age, marital status, alcohol intake, condom usage and more than one sexual partner in the past month. Results were reported with odds ratio (OR) with 95%CI. The overall model fit was assessed using the log likelihood ratio [2]. All data analysis was done using STATA version 13.

## Results

A total of nine hundred and fifty participants comprising 58% females and 42% males were enrolled (Table 1). The most frequent age group (42%) was 25–31 year-olds, 37% were married and a majority (96%) of them had some form of formal education. Overall, the proportion of participants who tested positive for either gonorrhea or chlamydia was 28% (95% CI: 24.8–30.6) and 11% (95% CI: 9.3–13.3), respectively. Males were more likely to test positive for gonorrhea and chlamydia than females (43.1% vs. 16.4%, *P* < 0.001) and (15.2% vs. 8.2%, *P* < 0.001) respectfully. Eighty-nine (25.3%) married participants tested positive for gonorrhea, while 174 (29.1%) unmarried participants tested positive for the infection. Chlamydia rates were higher among unmarried participants (13.0%) than married participants (8.2%) which was statistically significant (*p* = 0.03). A total of 31 participants had co-infections with these two pathogens, out of which 81% were males and 29% females.

**Table 1:** Demographic and Social behavioral Characteristics of Patients with Gonorrhea (GC) or Chlamydia (CT)

Variable	Male					Female				
	Tested (*N* = 401)	GC + ve n(%)	*P*-value	CT + ve n(%)	*P*-value	Tested (*N* = 549)	GC + ve n(%)	*P*-value	CT + ve n(%)	*P*-value
Age group(years)^a^			0.033		0.119			0.500		0.078
18–24	103	43(41.8)		11(10.7)		153	28(18.3)		19(12.42)	
25–31	177	86(48.6)		33(18.6)		226	40(17.7)		20(8.9)	
32–38	71	31(43.7)		13(18.3)		113	17(15.0)		5(4.4)	
39–45	30	6(20.0)		4(13.3)		41	4(9.8)		1(2.4)	
46 and above	18	5(27.8)		0(0.0)		16	1(6.3)		0(0.0)	
Marital status			0.767		0.748			0.537		0.036
Single	289	126(43.6)		45(15.7)		309	48(15.5)		33(10.4)	
Married	112	47(42.0)		16(14.3)		240	42(17.5)		13(5.4)	
Educational level			0.873		0.234			0.496		0.114
No education	15	7(46.7)		0(0.0)		23	4(17.4)		0(0.0)	
Primary/JHS	66	27(40.9)		9(13.6)		95	19(20.0)		12(12.6)	
Secondary and above	320	140(43.4)		52(16.3)		431	67(15.6)		33(7.7)	
Alcohol intake			0.021		0.344			0.511		0.308
Yes	180	89(49.4)		24(13.3)		114	21(18.4)		12(10.5)	
No	221	84(38.0)		37(16.7)		435	69(15.9)		33(7.6)	
Use of condoms^a^			0.455		0.471			0.052		0.538
Never	135	61(45.2)		19(14.1)		245	46(18.8)		24(9.8)	
Rarely	109	50(45.9)		13(11.9)		137	26(19.0)		11(8.0)	
On most occasion	75	34(45.3)		14(18.7)		73	9(12.3)		5(6.9)	
Always	74	26(35.1)		14(18.9)		66	4(6.1)		3(4.6)	
Having more than one sexual partner in the past month^a^			0.000		0.474			0.884		0.252
Yes	86	53(61.6)		11(12.8)		29	5(17.2)		4(13.8)	
No	314	104(45.4)		50(15.9)		512	83(16.2)		40(7.8)	

*GC* Gonorrhea, *CT* Chlamydia

^a^Variables with asterisk had missing responses, which ranged from *n* = 2 to 36

Regarding sexual practices of the participants, 380 (40.0%) reported never using condoms while 570 (60.0%) used a condom at least once. Among the 115 study participants (12.2%) who reported having more than one sexual partner in the past month, 82 (71.3%) tested positive for gonorrhea and 15 (13.0%) tested positive for chlamydia. Among males, there was a significant difference in gonorrhea rates between those who reported more than one sexual partner in the past month and those who had not, 62% versus 45% (*p* < 0.001). The rates of gonorrhea infections between females with multiple sexual partners (17%) compared to those who reported to have only one partner (16%) did not show any statistical significance. Additionally, there was no difference in the rates of chlamydia infections in men or women with multiple or only a single partner.

Almost a third of the study participants, 294 (30.9%) reported alcohol intake. Among those who drank alcohol, 200 (68.0%) drank at least once to five times a week, while 94 (32.0%) drank occasionally. Of those, 110 (37.4%) tested positive for gonorrhea and 36 (12.2%) for chlamydia.

Most participants presented with clinical symptoms such as discharge (85.3%), burning during urination (48.3%), and penile/vaginal pain (27.9%) (Table 2). Among males, statistically significant symptoms associated with gonorrhea infection were penile discharge, dysuria and hematuria. However, none of the symptoms was associated with chlamydia for men. Among females, none of the symptoms was significantly associated with gonorrhea or chlamydia.

**Table 2:** Clinical Presentation of Patients with Gonorrhea and Chlamydia

	Male					Female				
	Total (*N* = 401)	GC + ve n(%)	*P*-value	CT + ve n(%)	*P*-value	Total (*N* = 549)	GC + ve n(%)	*P*-value	CT + ve n(%)	*P*-value
Variable										
Painful Urination			0.001		0.283			0.856		0.724
Yes	238	119(50.0)		40(16.8)		221	37(16.7)		17(7.7)	
No	163	54(33.1)		21(12.9)		328	53(16.2)		28(8.5)	
Discharge			0.000		0.270			0.952		0.146
Yes	321	153(47.7)		52(16.2)		489	80(16.4)		43(8.8)	
No	80	20(25.0)		9(11.3)		60	10(16.7)		2(3.3)	
Pain in penis or vagina			0.767		0.219			0.813		0.378
Yes	112	47(42.0)		21(18.8)		153	26(17.0)		10(6.5)	
No	289	126(43.6)		40(13.8)		396	64(16.2)		35(8.8)	
Foul smell			0.767		0.763			0.544		0.101
Yes	67	30(44.8)		11(16.4)		161	24(14.9)		18(11.2)	
No	334	143(42.8)		50(15.0)		388	66(17.0)		27(7.0)	
Painful sex			0.640		0.764			0.474		0.629
Yes	23	11(47.8)		4(17.4)		107	20(18.7)		10(9.4)	
No	378	162(42.9)		57(15.1)		442	70(15.8)		35(7.9)	
Bleeding from Penis or vagina			0.024		0.501			0.424		0.647
Yes	12	9(75.0)		1(8.3)		60	12(20.0)		4(6.7)	
No	389	144(42.2)		60(15.4)		489	78(16.0)		41(8.4)	
Genital itching			0.304		0.527			0.392		0.833
Yes	42	15(35.7)		5(11.9)		129	18(14.0)		10(7.8)	
No	359	158(44.0)		56(15.6)		420	72(17.1)		35(8.3)	

In the multiple logistic regression, the factors which were significantly related to gonorrhea infection, but not chlamydia, among men included multiple sexual partners in the past month, dysuria and penile discharge (all *P*-values < 0.05). For females, use of condoms during sexual intercourse was the only factor significantly associated with gonorrhea. For women, a combination of these symptoms or demographic variables did not have any statistically significant association either. Females who reported that they or their partners always used condoms during sexual intercourse were less likely to test positive for gonorrhea, as compared to those who never used it (OR, 0.3; 95% CI, 0.1–0.8) (Table 3). Also, females who were married were less likely to test positive for chlamydia than those who were not married (OR, 0.4; 95% CI, 0.2–0.9) (Table 4).

**Table 3:** Multiple Logistic regression of gonorrhoea with selected patient characteristics

	Male Model, *N* = 398	
	odds Ratio(95% CI)	*P*-value
Age group(years)		
18–24	1(Ref)	
25–31	1.3(0.8–2.2)	0.343
32–38	1.1(0.6–2.0)	0.837
39–45	0.4(0.1–1.1)	0.069
46 and above	0.6(0.2–2.1)	0.462
Alcohol intake		
Yes	1.4(0.9–2.2)	0.106
No	1(Ref)	
Having more than one sexual partner in the past month		
Yes	2.3(1.3–3.8)	0.002
No	1(Ref)	
Painful urination		
Yes	2.1(1.4–3.3)	0.001
No	1(Ref)	
Discharge		
Yes	2.3(1.3–4.2)	0.005
No	1(ref)	
Bleeding from Penis or Vagina		
Yes	3.0(0.8–12.1)	0.117
No	1(ref)	
Female Model, *N* = 521		
Use of Condom		
Never	1(ref)	
On most occasions	0.6(0.3–1.3)	0.189
Always	0.3(0.1–0.8)	0.015

**Table 4:** Multiple logistic regression of chlamydia against selected patient characteristics

	Female Model, *N* = 500	
	odds Ratio(95% CI)	*P*-value
Age group(years)		
18–24	1(ref)	
32–38	0.5(0.2–1.4)	0.156
Marital status		
Married	0.4(0.2–0.9)	0.032
Use of condom		
Never	1(ref)	
On most occasion	0.5(0.2–1.4)	0.197
Always	0.3(0.1–1.1)	0.078
Having more than one sexual partner in the past month		
Yes	2.3(0.7–7.3)	0.159
No	1(ref)	

## Discussion

In Ghana, the existing published data on gonorrhea and chlamydia prevalence is mainly from women attending gynecological, antenatal or postpartum clinics [17–20]. Some of these studies found higher chlamydia prevalence than gonorrhea, contrary to results obtained in this study [21, 22]. The lower previously published infection rates of 0–15% for gonorrhea and 1.5–7% for chlamydia, could be attributed to different diagnostic methodologies employed and varied patient populations examined in this work. One major difference between our study and others includes using a more sensitive method, NAAT, to diagnose infection. Our study population contained 42% men who accounted for 66% of the gonorrhea and 58% of chlamydia cases. While women, who made up 58% of the study population, accounted for just 34% of the gonorrhea and 42% of the chlamydia cases.

As part of syndromic management, an assessment of risks based upon symptoms as well as demographic, and socioeconomic factors, aids in the clinical detection of STIs and helps direct limited resources to target individuals that are most at risk of having gonorrhoea or chlamydia. In this study, painful urination (50%) and urethral discharge (48%) were significantly associated with a diagnosis of gonorrhoea but not chlamydia in men. Therefore, our study results confirm that the syndromic approach may be better if utilized in health centres catering to symptomatic males to diagnose and treat gonorrhoea. Educating the general public on the possible symptoms associated with gonorrhoea would enable those at risk to seek early treatment. However, unlike the correlation observed between symptoms and positive diagnoses for gonorrhoea, we failed to identify any correlation between symptoms and chlamydia infection. A lack of an association with commonly correlated symptoms in the literature, especially for chlamydia could be due to a lack of power since only 15% of the males and 8% of the females had a positive NAAT. Perhaps other non-clinical risk factors that are behavioural or epidemiological in nature such as sexual preference, socioeconomic position, exchange sex for money, drug use, and sex with partners abroad would be relevant to improving chlamydia case detection [23].

**Fig. 1:**
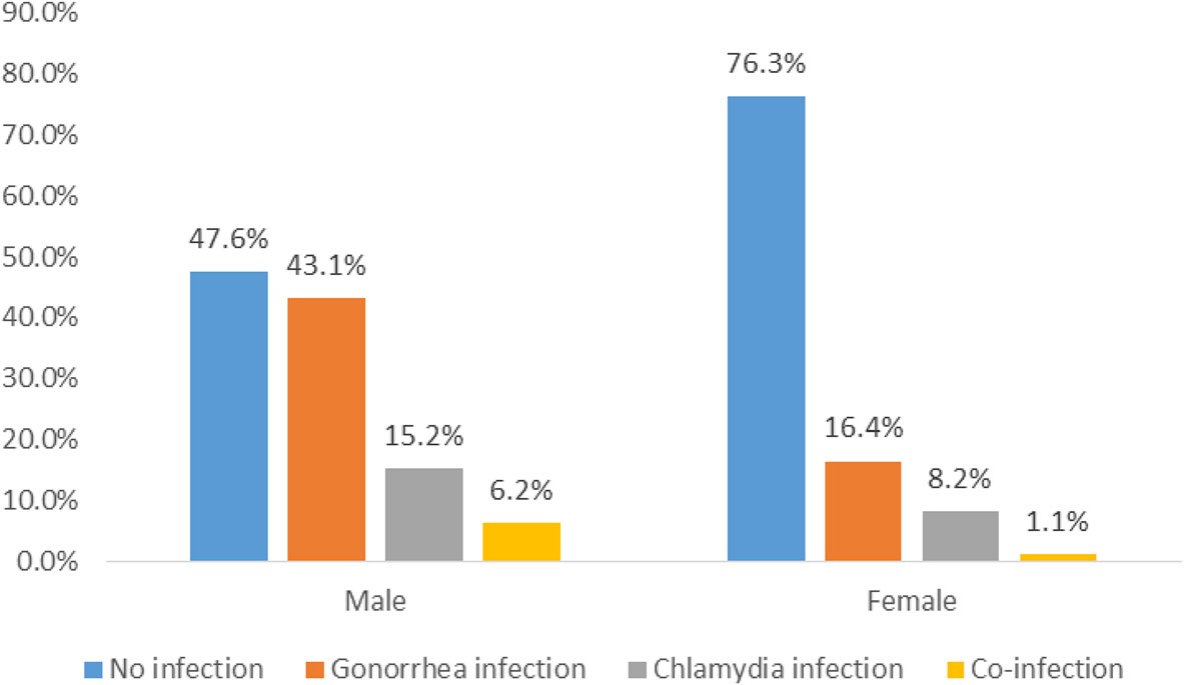
Distribution of Gonorrhea and Chlamydia infection status by Gender

Although all participants reported some symptoms that could be consistent with STIs, 47.6% of males and 76.3% of females who presented did not test positive for either disease (Fig. 1). Potential causes other than gonorrhoea or chlamydia infection include infectious aetiologies (e.g. trichomoniasis, candidiasis, *mycoplasma genitalium*) or non-infectious aetiologies. Given the high sensitivity of the NAAT assay, we speculate that there were likely few false negative tests. Since almost half the men and three-quarters of the women had no symptoms at all, a broad differential diagnosis should be considered by providers caring for patients presenting with symptoms consistent with a STI. Ideally, broad testing to include NAAT for GC and CT as well as for other potential aetiologies such as microscopy, vaginal pH assessment, or *mycoplasma genitalium* NAAT would be used if resources are available. This will avoid empiric therapy which exposes the patient to unnecessary antibiotics while fostering resistance among other pathogens. However, often time this is not the case and providers will need to consider empiric therapy for other aetiologies other than GC and CT.

Our survey identified three significant risk factors associated with higher rates of STIs: engaging in multiple sexual partnerships (MSP), inconsistent condom use, and alcohol intake. MSPs, defined as greater than one partner in the past month, are a common practice in certain areas in Ghana, predominantly among males [12]. A greater number of males (21%) were more likely to report engaging in MSPs than females (5%) from this study. This difference could reflect social desirability bias with men more likely to report having multiple sexual partners than women. Men with multiple sexual partners had significantly higher prevalence of (62% vs. 45%) gonorrhea infection compared to those who did not (*p*-value < 0.001).

The second significant risk factor associated with higher prevalence of gonorrhea was frequency of condom use in women with prevalence of 19% among those who never used condoms, 17% for inconsistent use of condoms and 6% for those who always used them. Females who always or even occasionally endorsed using condoms during intercourse have a lower likelihood of having gonorrhea as compared to those who never used condoms (OR, 0.3; 95% CI, 0.1–0.8). There was no statistically significant relationship between frequency of condom use and prevalence of either gonorrhea or chlamydia in men. Of note, 35% of men who stated they always use condoms were still positive for gonorrhea compared to a higher rate of 45% in those who never used condoms and the same held true for chlamydia with rates of 19% compared to 6%. The high prevalence of gonorrhea identified in males despite a reported compliance with condom usage may reflect recall or social desirability bias. We were unable to pursue repeat evaluations with the respondents that could have helped confirm the validity of the responses and believe this lack of statistical significance is due to the above biases and a lack of power, especially for chlamydia infections.

The third significant risk factor associated with higher rates of gonorrhea was alcohol use. Similar to others studies, men who reported consuming alcohol in this study were more likely to be infected with gonorrhea (49%) than those who did not (38%) [24]. Additionally, these individuals had a higher proportion of MSPs than those who did not consume alcohol (22.5% versus 7.5%, p<0.001). This finding supports a previously identified association between alcohol intake and MSPs [25]. A person’s judgment can be altered under the influence of alcohol, leading to high-risk sexual behaviors and is a known risk factor for contracting STIs in Africa [26]. Our data supports an association between alcohol intake and having multiple sexual partners leading to high-risk sexual behavior and the acquisition of a gonorrhea infection among males (*p* = 0.021).

Our data identifies three important risk factors to target in STI prevention efforts and to integrate into educational campaigns and clinical history-taking. The association between MSPs, alcohol intake and condom use with higher prevalence of gonorrhea make them high-yield information to obtain while risk stratifying patients who present with symptoms consistent with STI in Ghana. A simple survey instrument composed of just few questions could be easily executed during a clinic visit and help inform subsequent diagnostic testing. Also, they are potential topics for public health campaigns to change behavior among men and women at high risk for STIs.

## Conclusions

Despite the convenience sampling in referral clinics, we have identified a high prevalence of gonorrhea in Ghanaian males as well as risk factors for its acquisition, filling an important gap in knowledge.

Common symptoms significantly associated with gonorrhea among men in Ghana include penile discharge and dysuria. However, none of the common symptoms was significantly associated with chlamydia infection in men. There was no significant association between gonorrhea or chlamydia infection in women and common symptoms. MSPs and alcohol use were statistically associated with higher rates of gonorrhea in males while only the frequency of condom use was associated with gonorrhea in females. None of the risk factors, except marital status was associated with symptomatic chlamydia infection. These risk factors could be elicited upon evaluation of patients by a provider using a simple four question survey to help risk stratify them, better informing a syndromic treatment approach to STIs. The high-risk groups presenting with these risk factors represent a key focus area that needs to be identified by providers for diagnosis and treatment as well as for public health education campaigns.

## Limitations of study

- This study did not assess for other STIs and other culturally sensitive risk factors such as sexual preference, socioeconomic position, drug use, exchanging sex for money and sex with partners abroad.
- We did not conduct a systematic investigation into the prevalence of either chlamydia or gonorrhea in Ghana. Therefore we are unable to assert actual prevalence figures since by the studies design, it excluded infected but asymptomatic men and women.
- We acknowledge a likelihood of significant recall and social desirability bias inherent in studies looking at STI risk factors that could have influenced our results.

## Strengths of study

- Access to both men and women from five sites. There is a paucity of published information on gonorrhea and chlamydia rates in Ghana, especially among men. Published work on these STIs tend to be gender-biased, with more focus on females than males [20, 21, 27].

We used a more sensitive, specific, and less invasive method (urine NAATs) than has previously been used for diagnosis of gonorrhea (culture and gram stain) and chlamydia.

## Abbreviations

CI: Confidence interval
CT: *Chlamydia trachomatis*
DALYs: Disability Adjusted Life Years
DNA: Deoxyribonucleic Acid
GC: *Niesseria gonorrhoeae*
LR: Likelihood ratio
MSPs: Multiple Sexual Partners
NAAT: Nucleic Acid Amplification Testing
OR: Odds ratio
PID: Pelvic Inflammatory Disease
STIs: Sexually Transmitted Infections
